# A panoramic view of personalization based on individual differences in persuasive and behavior change interventions

**DOI:** 10.3389/frai.2023.1125191

**Published:** 2023-09-29

**Authors:** Alaa Alslaity, Gerry Chan, Rita Orji

**Affiliations:** Faculty of Computer Science, Dalhousie University, Halifax, NS, Canada

**Keywords:** personalization, persuasive technology, adaptation, user modeling, individual differences

## Abstract

Persuasive technologies are designed to change human behavior or attitude using various persuasive strategies. Recent years have witnessed increasing evidence of the need to personalize and adapt persuasive interventions to various users and contextual factors because a persuasive strategy that works for one individual may rather demotivate others. As a result, several research studies have been conducted to investigate how to effectively personalize persuasive technologies. As research in this direction is gaining increasing attention, it becomes essential to conduct a systematic review to provide an overview of the current trends, challenges, approaches used for developing personalized persuasive technologies, and opportunities for future research in the area. To fill this need, we investigate approaches to personalize persuasive interventions by understanding user-related factors considered when personalizing persuasive technologies. Particularly, we conducted a systematic review of 72 research published in the last ten years in personalized and adaptive persuasive systems. The reviewed papers were evaluated based on different aspects, including metadata (e.g., year of publication and venue), technology, personalization dimension, personalization approaches, target outcome, individual differences, theories and scales, and evaluation approaches. Our results show (1) increased attention toward personalizing persuasive interventions, (2) personality trait is the most popular dimension of individual differences considered by existing research when tailoring their persuasive and behavior change systems, (3) students are among the most commonly targeted audience, and (4) education, health, and physical activity are the most considered domains in the surveyed papers. Based on our results, the paper provides insights and prospective future research directions.

## 1. Introduction

Persuasive Technologies (PTs) are interactive systems designed to motivate behavior change (Fogg, 2003). PT has been used in many different application domains. For example, managing online gambling habits (Arden-Close et al., 2022), motivating people to make stronger passwords in the domains of computer security (Forget et al., 2008), promoting healthy behavior (Aldenaini et al., 2020), and increasing users' engagement with eCommerce applications (Alslaity and Tran, 2021). Since the emergence of PT in the early 2020s (Fogg, 2002), most existing PT have adopted a “one-size-fits-all” approach (Busch et al., 2015). However, there has become a consensus in the literature that the one-size-fits-all design of PT is inadequate to promote the desired outcome and could reduce the effectiveness of the intervention (Adnan et al., 2012; Alslaity and Tran, 2021). This consensus has led to a growing interest in finding ways to personalize and tailor PTs and considerable research has investigated how to better design PT to increase motivation and the probability of success (Andrew et al., 2007; Fogg, 2009; Brynjarsdóttir et al., 2012; Weiser et al., 2015; Jalowski et al., 2019; Aldenaini et al., 2020).

The realization of the importance of personalizing PTs has led to an increasing number of studies investigating various ways that PTs can be personalized and relevant personalization dimensions. These studies considered different personalization approaches and adopted various adaptation techniques with the aim of increasing the effectiveness of their PTs to motivate behavior change. For example, in the context of motivating healthy eating behaviors, Orji et al. (2014) investigated the effects of personalizing persuasive games interventions on different gamer types. The researchers found that Competition and Comparison are perceived as persuasive and receptive for five gamer types (Conquerors, Masterminds, Seekers, Socializers, and Survivors), but negatively perceived by Daredevils. The researchers also found that Praise is appealing for Seekers but was perceived negatively by Socializers. In the domain of health and physical activity, Schoeppe et al. (2017) recommend that future design and development of apps that promote health behavior change should be personalized and tailored to the target audience and informed by evidence-based health behavior guidelines and theories.

Further work in the education domain (Orji et al., 2019a) demonstrates that using a single strategy is more effective in motivating students and keeping them engaged than incorporating multiple random strategies if the single strategy is tailored to a particular user group. Other research in the eCommerce and recommender systems domain found that personalizing persuasive cues to users' personality traits would enhance users' engagement and acceptance of recommendations (Alslaity and Tran, 2021). In a recent review, Anagnostopoulou et al. (2018) examined existing literature on persuasive system implementations in the context of sustainable mobility and found that personalization is an important attribute to improve the impact of the systems and increase the acceptability for real-life usage (e.g., route suggestions based on energy consumption).

Although the research in personalizing persuasive technologies is gaining increasing attention, there is a lack of reviews that summarize the trends and latest technologies in the domain. Therefore, it becomes essential to conduct a review to synthesize available literature. To fill this need, we conducted a systematic literature review to provide an overview of the current trends, challenges, and techniques for developing personalized persuasive technologies. Particularly, we systematically analyzed 72 papers published in the last ten (10) years. The reviewed papers were retrieved from ACM, IEEE, Scopus, and PubMed digital libraries; then, they were evaluated against various themes: metadata, technology, target outcome, individual differences, theories and scales, and evaluation approaches.

Personalization is a broad topic adapted to different domains, including recommender systems, health interventions, and persuasive technologies. Moreover, personalization can be achieved based on various factors, including user-related factors (user characteristics), system-related factors, and contextual factors. This multidimensionality of personalization approaches and the wide spread of personalization over several domains makes it hard to cover the whole topic in a single research paper; therefore, this systematic review focuses on personalization in persuasive technology, and it only considers personalization of persuasive technology based on users-related factors, while excluding personalization based on other factors (e.g., system logs and contextual factors). User-related factors are characteristics that are specific to individuals. These factors can vary from person to person and can include demographics, stages of change (Mulchandani et al., 2022), personality traits (Alslaity and Tran, 2020a; Ghorbani and Semiyari, 2022), cognitive ability, gamer types (Tondello et al., 2016), and culture. It is worth mentioning that culture is a broad concept that includes an individual's behaviors, ethics, beliefs, norms, and habits., etc. That is, part of the culture is related to an individual's characteristics. Also, some researchers use the word “culture” to indicate countries, which is part of individual's demographics. So, we considered it as part of the user-related characteristics.

This review aims to (1) provide a broad overview of the personalization factors considered in persuasive interventions, (2) highlight the emerging trends and popular domains concerning technological interventions, personalization, and individual differences, and (3) summarize the limitations of existing personalized persuasive technology interventions. Our results show that (1) within the past 10 years, there has been increased attention toward personalizing persuasive interventions, (2) personality traits are the most popular dimensions to investigate individual differences, (3) students are among the most commonly targeted audience, and (4) education, health, and physical activity are the most heavily researched and considered domains in the surveyed papers. Based on our results, the paper provides insights and prospective future research directions.

The remainder of this paper is organized as follows. Section 2 discusses the background and related work. Section 3 presents the methodology, followed by the results in Section 4. Section 5 discusses the findings and provides future research directions, while Section 6 concludes the paper.

## 2. Background

In this section, we briefly introduce the main concepts and background knowledge, including Persuasive Technology, Personalization, and Gamification. Then we identify the scope of the study.

### 2.1. Persuasive technology

Persuasion is defined as a style of communication designed to influence the actions and behaviors of individuals (Jones and Simons, 2017). It is inherent in human nature, and it involves the *subject* (or Persuader) and *object* (or Persuadee). The advent of modern interactive devices, such as handheld devices, mobile devices, and sensing and tracking devices, opened the doors for a techno-dependent form of persuasion called *Persuasive Technology*. In comparison, Persuasive Technology (PT) can be defined as the use of technology to influence the behavior and the attitude of users (Oinas-Kukkonen and Harjumaa, 2008). PT aims to bring desirable changes in attitudes and behaviors without using deception, coercion, or inducements. PT has been shown to be effective at motivating behavior change in many different domains including health and wellness (Orji and Moffatt, 2018), education (Orji et al., 2019b), fitness and physical activity (Matthews et al., 2016; Oyebode et al., 2021a), sustainability (Knowles et al., 2014), and eCommerce (Adaji, 2017). These technologies have been shown to be effective at promoting several desired behavior change goals including motivating behavior change, attitude change, motivation and engagement, compliance, and increasing awareness.

The design of persuasive interfaces involves using persuasive strategies designed as techniques used to motivate behavior change and achieve desired goals, such as increasing engagement, adoption, continued use, and increase awareness. Over the years, researchers have proposed various persuasive strategies over the years to achieve various behavior objectives. Among the most common persuasive strategies are Fogg's seven persuasive technology tools (Fogg and Fogg, 2003), Cialdini's six weapons of influence (Cialdini, 2001), and the Persuasive Systems Design (PSD) Model (Oinas-Kukkonen and Harjumaa, 2009), which proposes 28 persuasive strategies.

Since the introduction of persuasive technologies in the early 2020s (Fogg, 2002), the majority of persuasive technologies have typically treated all users in a similar manner without taking into account users' differences (Alslaity and Tran, 2020b, 2021; Mulchandani et al., 2022). However, some research indicates that the effectiveness of PTs can vary among different users. This is because individuals are unique and have different characteristics such as demographics and personality traits, can impact their behavior. As a result, it is widely recognized in the literature that the one-size-fits-all approach is inadequate in achieving the desired outcomes and may hinder the effectiveness of PTs (Moher et al., 2009; Gamberini et al., 2012). Consequently, there is a growing interest in customizing and personalizing persuasive interventions and adapt based on user characteristics and preferences to enhance their effectiveness (Drozd et al., 2012). The next section discusses the personalization concept and how PTs can be personalized.

### 2.2. Personalization

Personalization is a method where systems tailor their contents to individual preferences (Forget et al., 2008). Fan and Poole (2006) discuss the personalization techniques in the domain of information systems and find that personalization can be classified based on multiple aspects. One aspect relates to *what can be personalized*, such as the information itself (content), how the information is presented (user interface), and what users do with the system (functionality). Another aspect is *the target of personalization*—whether the personalization is for a specific individual or a group of individuals. A third aspect is the idea of s*ystem-driven* vs. *user-driven* personalization (Orji et al., 2017b). System-driven (or implicit) personalization is when the personalization is driven by the system. In contrast, user-driven (or explicit) personalization is initiated by the user, where they make choices or provide information to guide the system on how to adapt.

As mentioned previously, most existing PT adopt a “one-size-fits-all” approach (Busch et al., 2015) in their design and evaluation, reducing their effectiveness at motivating desired behavior change. However, there is a consensus in the literature that personalization is paramount for designing persuasive systems because it has been shown to be more effective at motivating behavior change than the “one-size-fits-all” approach (Fogg, 2002; Göbel et al., 2010; Orji, 2016; Ouzzani et al., 2016). As a result, there is growing interest in finding ways to personalize and tailor PT to increase its effectiveness. Recent research has been focusing on how personalization can be effective. For example, Oinas-Kukkonen et al. (2022) argue that because a user's behavior can change over time, the grounds for personalization may change without the system detecting the change, which results in an “outdated” view of the user. Therefore, the more personalized the intervention, the more users relate to it and find it helpful, which will, in turn, increase the likelihood of adoption of the intervention.

Persuasive interventions have been personalized mainly based on user characteristics such as personality (Jankovič et al., 2022), gamer type (Orji et al., 2018a), cultural orientation (Orji, 2016), individual attitudes (Teeny et al., 2021), and users' demographics. For example, research shows that individuals who score high in extraversion prefer socially competitive activities such as points, levels, and leaderboards (Jia et al., 2016), while another study shows that personalizing features and adapting the gameplay to a player's stress levels, duration and intensity can enhance a player's motivation toward gameplay (Göbel et al., 2010). Furthermore, personalizing persuasive exercise training systems based on the player's competitiveness and cooperativeness can create a highly enjoyable experience motivating the player to continue the activity (Shaw et al., 2016).

Personalization techniques can be divided into three categories: static, dynamic, and hybrid. Static personalization approaches involve the use of fixed rules to personalize the system. In these approaches, a correlation is established between user models (information gathered about the target users) and one or more personalization rules, creating a mapping between users or user groups and the rules during the design phase. Each mapping signifies that the system should apply a specific rule to personalize the intervention when a user's current state aligns with the criteria defined in the mapping. The main advantage of static approaches is their simplicity. They are easy to be implemented. However, static approaches fail to obtain the ever-changing users' profiles and preferences.

On the other hand, in dynamic personalization, the user model undergoes continuous updates as the user interacts with the intervention. Consequently, the intervention actively monitors changes in the user model and adjusts the system accordingly. For instance, a persuasive intervention for promoting physical activity might use the reminder strategy with the user who is in the early stages of doing exercise. Concurrently, the intervention keeps track of the user's progress, such as step count and exercise duration. Once the user surpasses a predefined threshold, the user profile is updated, indicating an increased level of physical activity. As a result, the system adapts by employing a more appropriate persuasive strategy, such as competition, that aligns with the user's current stage. To achieve dynamism, designers can identify a range of interaction styles or scenarios for different user models. This identified information is then mapped to relevant adaptation rules, enabling the system to adapt dynamically based on user characteristics and contextual factors. A more robust approach to dynamic adaptation is to machine learning (ML) techniques are used to model user knowledge, characteristics, preferences, and goals, as well as interactions between the user and the system. In this approach, an ML model is initially trained on both user data and interaction (contextual or behavioral) data; hence able to infer individual users' current situations and interaction patterns and predict a user's next action or behavior.

Although dynamic personalization approaches overcome the drawbacks of static approaches, they are not the ideal solution for all cases. For example, new users' profiles may lack a sufficient amount of data (known as the cold-start problem), making it hard to use these profiles with ML models. This calls for a hybrid approach that combines static and dynamic approaches. In such cases, static personalization is used whenever dynamic personalization is not applicable (e.g., the cold-start case).

### 2.3. Persuasive technology, gamification, and games

This section discusses the relationship between persuasive technologies, gamification, and games. Broadly, “gamification” refers to the application of game principles in non-game contexts (Deterding, 2012). The term gamification has also been defined as “active ingredients” to make games addictive, and to apply gamification well, developers first need a list of game design elements followed by integrating these elements into their intervention. Gamified systems become persuasive when they employ specific behavior change ingredients encouraging people to shift their beliefs, attitudes and actions (Cugelman, 2013).

Persuasive technologies and gamification are interrelated domains. Stemming from Fogg's formulation of PT (Fogg, 2003), PT is applied to the function and design of gamification systems to describe the tool (an interactive product designed to motivate behavior change) (Llagostera, 2012). In return, gamification can be embedded into the design of persuasive systems to increase the effectiveness of the intervention. Several interventions have been proposed, where researchers have proposed interventions that combine gamification and PTs. For example, Barratt (2017) explored the impact of incorporating persuasive strategies and gamification elements on cycling practice. The study found that gamification helps cyclists to establish new patterns and regimes of cycling to motivate themselves to maintain and improve their health and fitness goals. Martin and Kwaku (2019) investigated the relationship between user types [using the HEXAD model (Tondello et al., 2016)] and persuasive principles in the context of energy saving gamified system (Barratt, 2017). Altmeyer et al. (2018) examined a gamified approach for designing mobile fitness applications. The researchers found that a gamified system that combines integrates gamification elements and persuasive strategies, such as journaling and social comparison, motivated users to walk more.

Games, on the other hand, are systems designed for entertainment. However, games can also be used for purposes other than entertainment (e.g., behavior change, education, and health). Games that are designed for purposes other than entertainment are commonly known as serious games (Altmeyer et al., 2021). Various studies argue that there is a growing interest in games promoting positive behavior change and that persuasive games (games that deploy persuasive technologies) have been exploited to tremendous effect with applications in various domains (Ghorbani and Semiyari, 2022; Mulchandani et al., 2022). As such, persuasive technologies have become an essential component of the modern game designer's toolkit when designing solutions to motivate behavior change. Several researchers have explored the feasibility of using games for promoting behavior change. For example, Mulchandani et al. (2022) explored the idea of using personalized persuasive games for increasing disease awareness. In a different study, Orji et al. (2017a) conducted a large-scale study of 660 participants to investigate the benefit and applicability of persuasive games in the health domain, and how these games can be personalized based on personality traits. The researchers found that conscientious individuals tend to be motivated by persuasive strategies such as goal setting, simulation, self-monitoring and feedback; whereas individuals who score high on openness to experience are more likely to be demotivated by rewards, competition, comparison, and cooperation.

To summarize, gamification and PTs can be used for behavior change and motivation (Martin and Kwaku, 2019). That is, gamified systems can be persuasive, and behavior change systems. For example, gamification elements can be incorporated as features in a learning management system to motivate students to keep up their good work. Also, games can be used to motivate behavior change. For instance, researchers have examined the use of games to motivate various behaviors, such as physical activity (Yim and Nicholas Graham, 2007; Mazeas et al., 2022), or to increase awareness toward particular risks (Mulchandani et al., 2022). To enhance the effectiveness of these serious games, they are incorporated with persuasive techniques. Accordingly, we can say that gamification and games can be used hand-in-hand with persuasive technologies. Persuasive technologies can be integrated with gamification techniques or in games design to enhance their effectiveness in promoting the desired goal.

## 3. Research method

This systematic review is conducted following the Preferred Reporting Items for Systematic Reviews and Meta-Analyses (PRISMA) guidelines for systematic reviews (Moher et al., 2009). Our goal is to systematically analyze personalization approaches used in designing PT for motivating behavior change.

The present review is guided by the following seven research questions:

RQ1 What are the trends in the research in the domain of personalization and PT?
RQ2 What are the personalization aspects used in persuasive interventions?
RQ3 How are individual differences captured in persuasive and behavior change interventions?
RQ4 What are the most common theories and motivational strategies used for developing and evaluating personalized PT?
RQ5 What are the most common challenges and limitations facing the domain of personalized and adaptive systems?
RQ6 What are the most common goals of personalized persuasive interventions?
RQ7 What are the limitations and future research directions in personalized persuasive interventions?

The process we adopted for conducting this review was guided by the Search, Appraisal, Synthesis and Analysis (SALSA) analytical framework proposed by Grant and Booth (Booth and Grant, 2009). The following are the details of each step.

### 3.1. Search

A comprehensive literature search was conducted in major computer science and social science bibliographical databases, including ScienceDirect, PubMed, the ACM Digital Library, and the IEEE Xplore Library. The search was based on the title, abstract, and keywords of the papers. We also searched using a combination of key terms, including adaptation, personalization, customization, persuasion, motivation, tailoring, and intervention. We also used logical operators (AND and OR) to cover the combination of different terms. The search was limited to papers published and combined the search query with filters for publication date (2012–2022) to limit the results to papers published in the last ten years. The search query used is provided in Appendix I. Our search query returned a total of (6,887) papers distributed as follows: the ACM digital library (3,759), IEEE Xplore (294), PubMed (34), and Scopus (2,800).

### 3.2. Appraisal

The researchers exported the results from each of the databases in a BibTeX format and uploaded to *Rayyan*, a free web and mobile app that helps with conducting research by expediting the screening process using a semi-automated process (Ouzzani et al., 2016). Rayyan helps to facilitate a group-based screening process, as well as finding and resolving duplicates. After uploading the documents, we excluded 63 duplicates. Then, we screened the papers against the inclusion/exclusion criteria. Below are the inclusion criteria used to filter papers. All included papers are:

- Peer-reviewed papers that discuss adaptation and personalization based on users' characteristics.
- Investigate persuasion and user's characteristics; whether the study includes the design and development of a persuasive intervention. That is, papers that investigate personalizing persuasive interventions based on questionnaires and a prototype or wireframe of an intervention are included.
- Introduce and evaluate a new PT, evaluate an existing PT, or discuss the design of PT in general
- are published in English, and
- published in the last ten years (2012–2022).

We excluded papers describing the design and development of PT without an evaluation component, position papers, review papers, and papers that discuss personalization but not regarding PT.

The screening process involved three steps:

1. Title screening: we screened the retrieved titles, where we retained papers with titles that seemed related to the area of interest. After screening based on the title, 691 articles were deemed relevant.
2. Abstract screening, where we assessed the paper against the inclusion/exclusion criteria. 116 were retained after filtering based on the abstract.
3. Full-text review; in this step, we evaluated the full paper text to make sure that we only retained papers that related to the domain and involved all the required information. After the full read of the papers, 44 more papers were excluded, and a total of 72 were included in this analysis.

**Figure 1:**
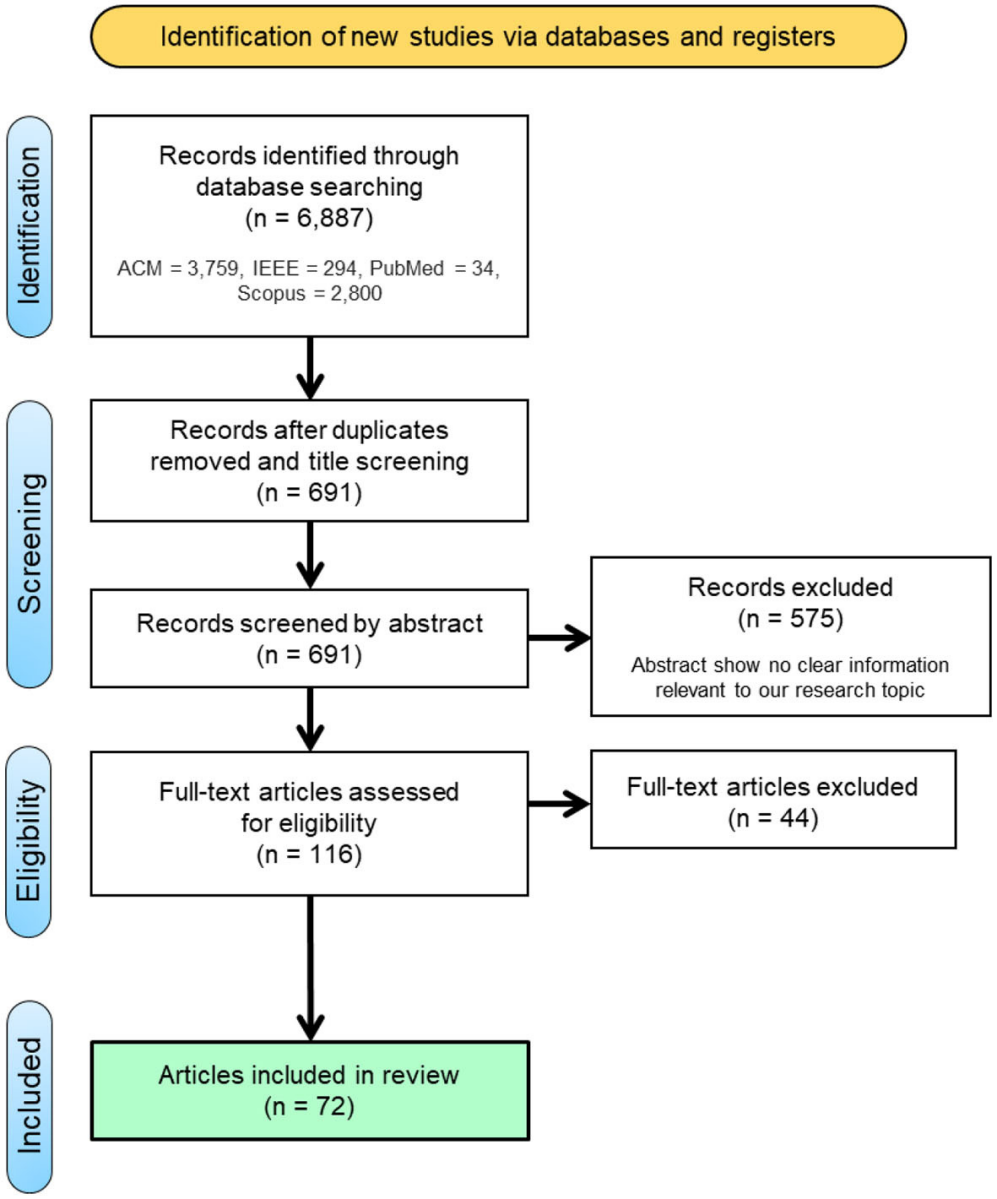
Included study identification process flow diagram.

Figure 1 summarizes the screening process. The screening process was done by two independent researchers using the Rayyan web app. The agreement rate was 93%. All conflicts were discussed between the researchers, and they reached an agreement on the final inclusion/exclusion decision. A flow diagram of the screening process is presented in Figure 1.

### 3.3. Synthesis

After filtering the papers, we reviewed and analyzed the retained papers. At this stage, we developed a coding sheet to summarize the relevant information inferred from the evaluated papers. Particularly, we extracted data related to the following themes:

- *Meta data*. Including the publication year, region, venue, and publisher.
- *Application domain*. Identify the domain of the presented research.
- *Technology*. The type of PT adopted or developed in the paper.
- *Personalization Aspect*. User characteristics used to inform personalization and the user profile.
- *Models, Scales, and Theories*. The models and theories used for user modeling and the scales used to evaluate personalized persuasive systems.
- *Motivation or behavioral change strategies*. The most common persuasive and behavior change strategies used in the evaluated papers.
- *Target outcome*. The goal of the PT discussed in the papers.
- *Evaluation approach*. The evaluation method, duration, target audience, and participants' age and gender.

The data collected under these themes will be used to answer the research questions, as follows: metadata, application domain, and technology (RQ1), motivation or behavioral change strategies (RQ2), personalization aspects (RQ3), models, scales, and theories, and evaluation approaches (RQ4), target outcome (RQ6), while RQ5 and RQ7 will be answered by the authors based on the results of the other research questions.

### 3.4. Analysis

Finally, we analyzed the data extracted in the previous step. We tabulated all the data and cleaned it. Then we conducted meta and descriptive analyses and calculated correlations between the variables. Data tabulation and analysis were done using Microsoft Excel. Two authors were involved in the data analysis.

## 4. Results

This section discusses the results obtained from the data analysis. The results are presented according to the themes discussed in Section 3.3. Before presenting the results, it is worth mentioning that not all results categories are mutually exclusive. For example, the same paper might have two different target outcomes. In such cases, we counted the paper under each category.

### 4.1. Meta-analysis

**Figure 2:**
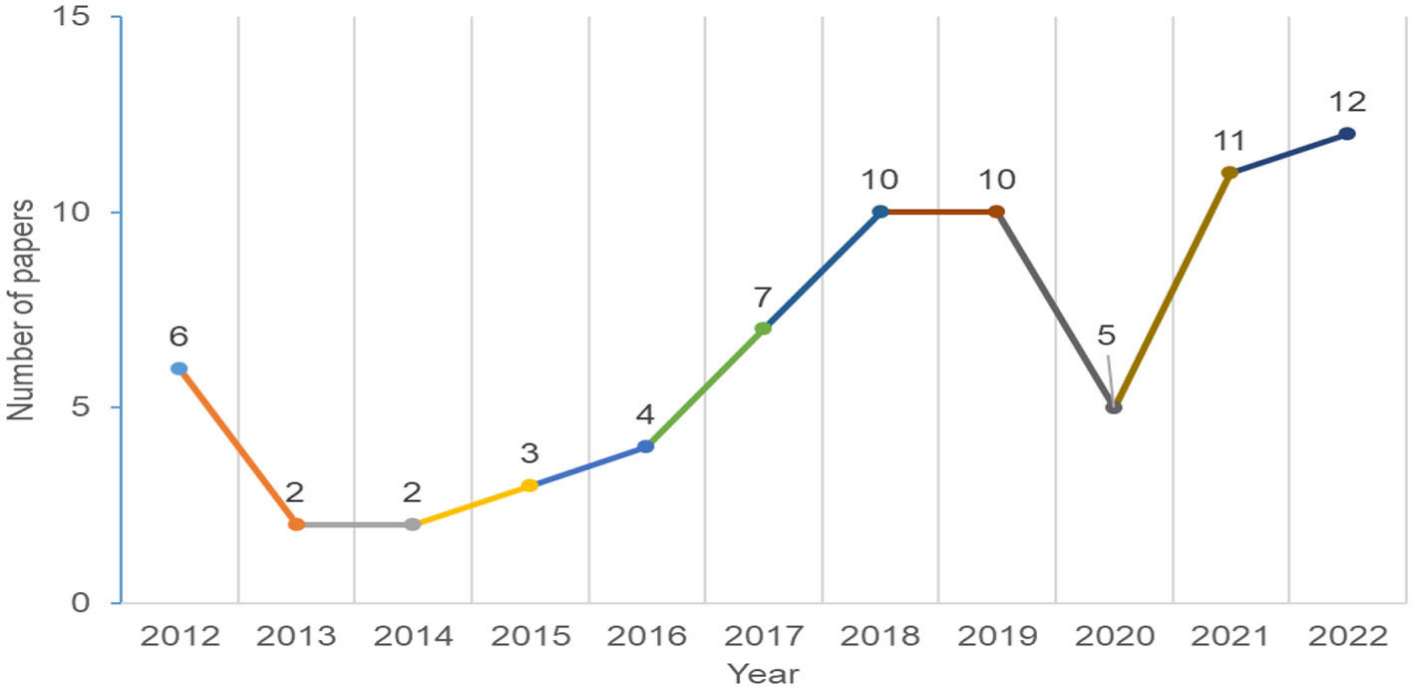
Distribution of papers by year.

We first analyzed metadata to provide an overview of the trends and publication venues. We analyzed data about the year of publication, publication venue, and publishers. Figure 2 shows publications by year. The figure shows an increase in the number of papers discussing personalization and persuasive technology since 2013, with one exception in 2020, where the number of papers dropped compared to the previous year. There is no confirmed reason for this drop, but it could be because of the COVID-19 pandemic, where all research slowed down. Nonetheless, Figure 2 shows a relatively constant increase in publications in the domain of personalized persuasive interventions.

**Figure 3:**
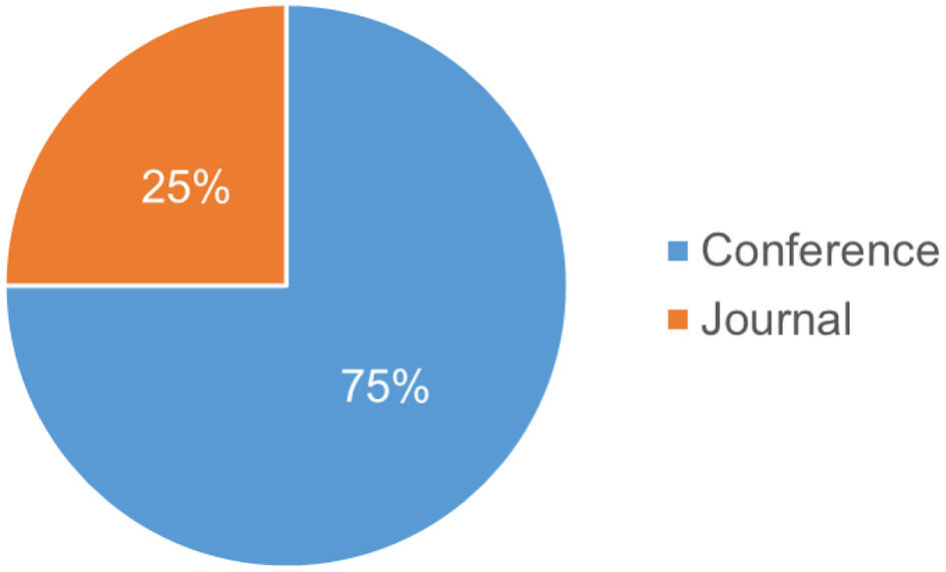
Distribution based on publication type.

Regarding the publication venues, our results show that most of the considered papers (75%) are published in conferences, while 25% were published in journals, as depicted in Figure 3. There is no clear and confirmed justification of these results.

**Figure 4:**
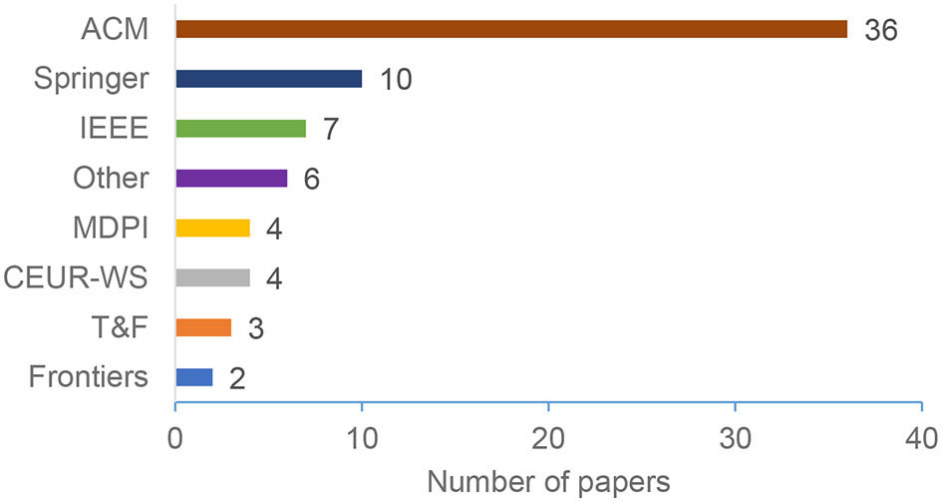
Distribution by publisher.

The considered papers were published in many different venues (e.g., International Conference on Persuasive Technology, International Conference on User Modeling, Adaptation and Personalization, and Conference on Human Factors in Computing Systems) distributed between (13) publishers. ACM emerged as the most common publisher, with (50%) of the papers published in one of ACM venues. Springer comes next, with (14%), followed by IEEE (10%). Figure 4 summarizes our results regarding the publisher's distribution. Table 1 summarizes the most common venues.

**Table 1:** Most common venues.

**Conference**	**Publisher**
International Conference on Persuasive Technology	Springer
International Conference on User Modeling, Adaptation and Personalization (UMAP)	ACM
Conference on Human Factors in Computing Systems (CHI)	ACM
International Conference on Artificial Intelligence in Education, AIED	Springer
Behavior and Information Technology	Taylor and Francis
JMIR mHealth and uHealth	JMIR

### 4.2. Application domain

**Figure 5:**
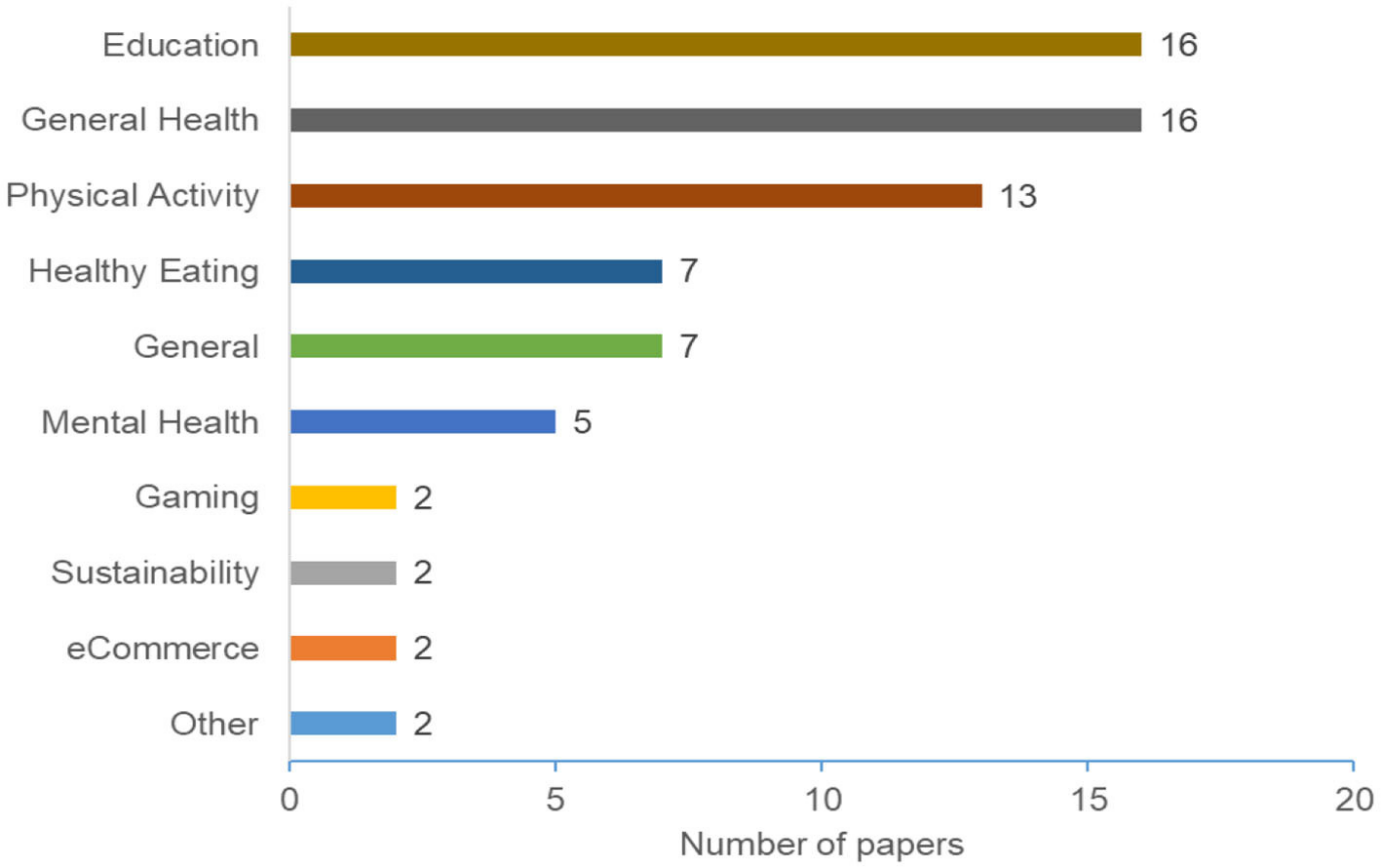
Distribution of application domains.

Our study revealed that the research in this area is focused on ten application domains. Figure 5 depicts the distribution of these application domains. The figure shows that education and general health are the most common domains. The general health category represents papers that consider the health domain without specifying a particular area. In addition to the General Health domain, several papers focused on a particular health-related domain, including Physical Activity (13 papers), Healthy Eating (seven papers), and Mental Health (five papers). Considering all health-related domains, we notice that a total of 41 papers (~57%) focused on the health and wellness domain.

Some papers discuss PT in general (i.e., without specifying a particular domain). We categorized these papers under the *General* category. The *Gaming* category indicates papers that discuss personalized PT in the gaming domain. This includes papers that adopt PT to motivate or change a gamers' behavior. For example, helping gamers achieve their desired gaming habits (Zhou et al., 2021). The physical activity domain, consist of research related to personalizing PTs to promote physical fitness or activity (Altmeyer et al., 2021). Table 2 summarizes the considered paper and their distribution based on application domain.

**Table 2:** Summary of papers by domain.

**Domain**	**References**
Education	Forget et al., 2008; Oinas-Kukkonen and Harjumaa, 2008; Cugelman, 2013; Nacke et al., 2014; O'Keefe, 2015; Adaji, 2017; Orji et al., 2017a, 2018a,b; Anagnostopoulou et al., 2018; Orji and Moffatt, 2018; Abdullahi et al., 2019b; Jankovič et al., 2022; Mulchandani et al., 2022
General health	Goldberg, 1999; Cialdini, 2001; Fogg and Fogg, 2003; Fogg, 2009; Oinas-Kukkonen and Harjumaa, 2009; Brynjarsdóttir et al., 2012; Deterding, 2012; Gardner et al., 2012; Orji et al., 2014, 2019a; Matthews et al., 2016; Altmeyer et al., 2018; Hofstede and Milosevic, 2018; Martin and Kwaku, 2019; Mazeas et al., 2022; Oinas-Kukkonen et al., 2022
Physical activity	McCrae and Costa, 1987, 1989; Bandura, 2002; Andrew et al., 2007; Midden et al., 2008; O'Keefe, 2013; Orji, 2016; Orji et al., 2017b, 2019b; Modic et al., 2018; Mora et al., 2019; Alslaity and Tran, 2020a
Healthy eating	Fogg, 2002, 2003; Goldberg et al., 2006; Knowles et al., 2014; Martin and Kwaku, 2019; Klock et al., 2020; Feroz et al., 2021
Mental health	Bassili, 1996; Booth and Grant, 2009; Barratt, 2017; Aldenaini et al., 2020
Gaming	Anagnostopoulou et al., 2019; Oyebode et al., 2021b
Sustainability	Abdullahi et al., 2018; Alslaity and Tran, 2021
eCommerce	Jalowski et al., 2019; Alqahtani et al., 2022
Others	Busch et al., 2015; Oyebode et al., 2021a

### 4.3. Personalization aspects

**Figure 6:**
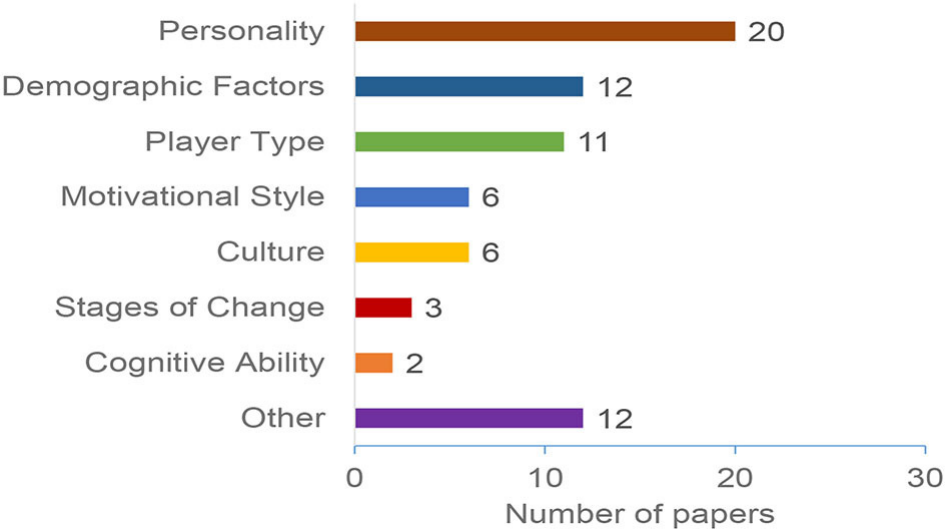
Distribution of papers by personalization aspects.

Personalized persuasive technologies explore various user characteristics to identify and account for differences between users. Examples of these characteristics include personality traits, age, gender, culture, and gamer types. In this section, we discuss the personalization aspects considered to distinguish users and build users' profiles that can be used for personalization. Our results revealed more than 10 different personalization aspects that are considered by research in this area. Figure 6 summarizes the most common aspects. As the figure shows, *Personality Traits* are the most popular and heavily studied user characteristics, followed by *Player Types* and *demographic factors*. The category “Other” includes the less common aspects, such as emotions, occupation, and learning style.

### 4.4. Scales, models, and theories

This section presents our results regarding the models, scales, and theories adopted in the considered papers. These scales and theories are used for different purposes, including identifying individuals' differences and users modeling, systems design, and evaluation.

**Figure 7:**
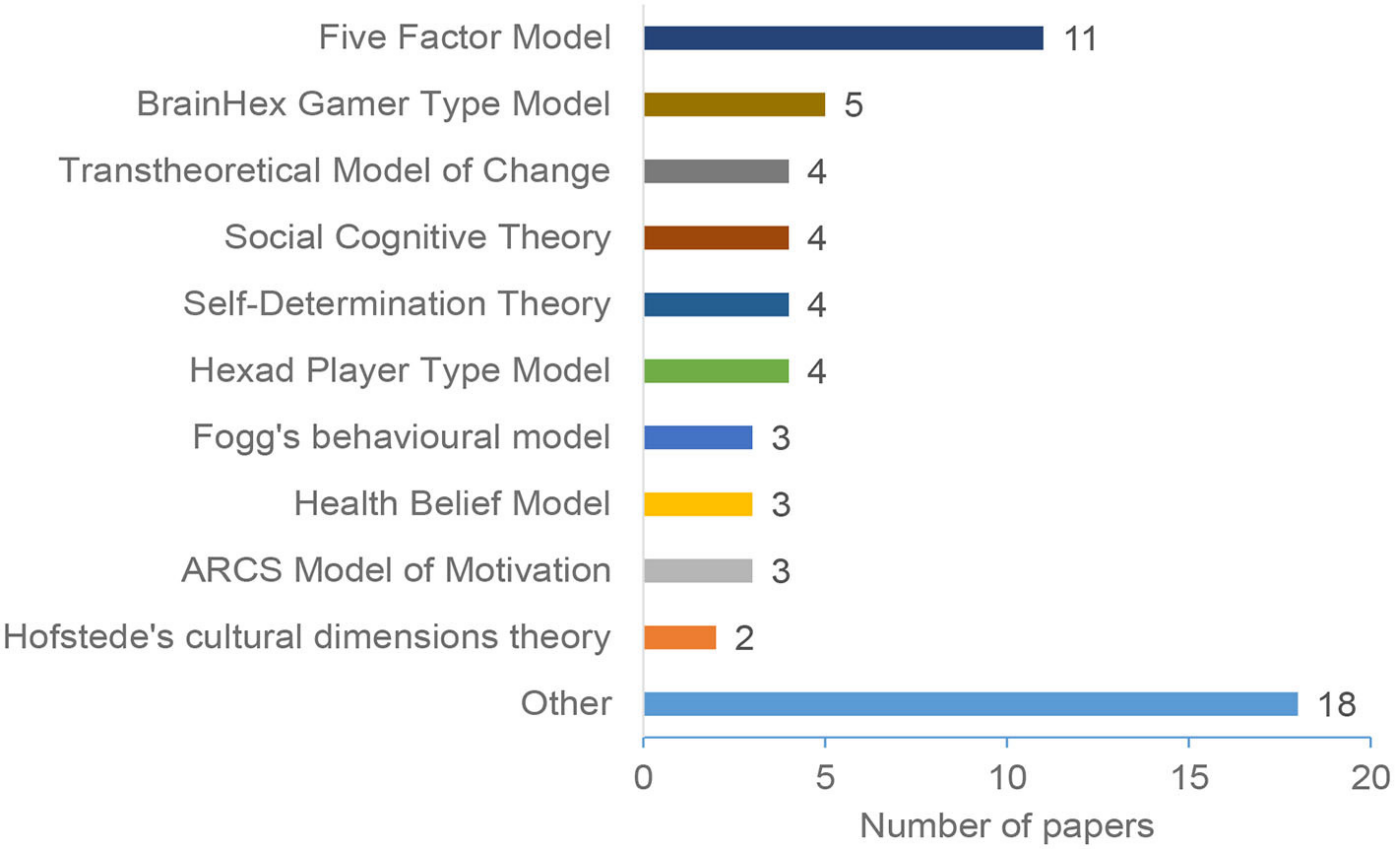
Models and theories.

Figure 7 depicts the most common theories and models used in the reviewed papers. These theories are mainly used to identify individual differences and, therefore, identify different user groups. As Figure 7 depicts, the Five Factor Model is the most commonly adopted model, followed by the BrainHex Gamer Type Model (five papers). Then the Transtheoretical Model, the Social Cognitive Theory, Self-determination Theory, and Hexad Players Type model are considered by four papers each. Our study identified 28 models and theories; however, Figure 7 only shows the most popular models. All other models are grouped under the “Others” category; Models and theories used in less than two papers are grouped under the “Other” category. Examples of these theories are: Honey and Mumford's learning styles (Anagnostopoulou et al., 2018), Index of Learning Styles (ILS) (Orji and Moffatt, 2018), Fear-Avoidance model (Cialdini, 2001), and Toulmin model of argumentation (Orji, 2016).

**Figure 8:**
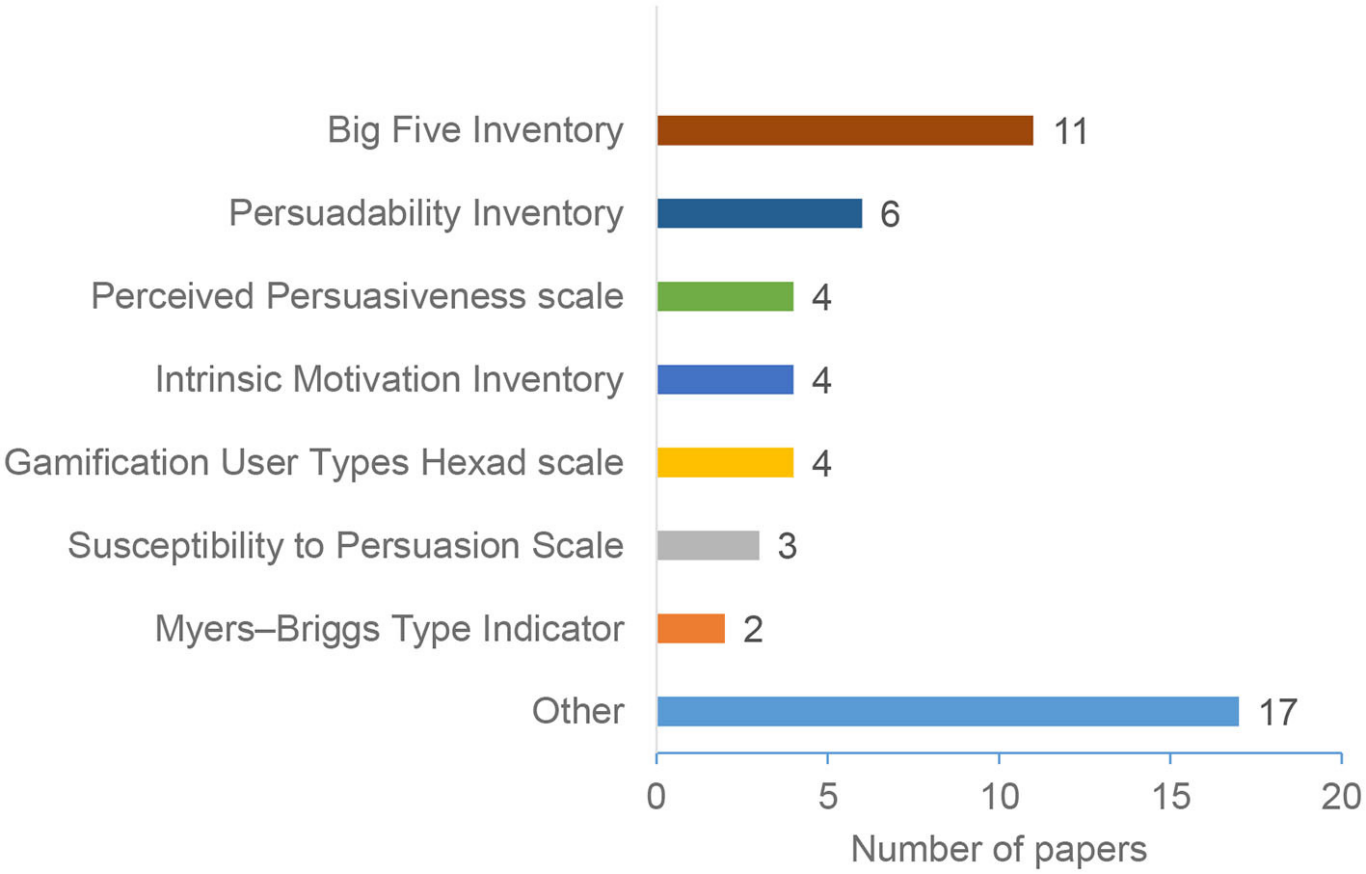
Scales and inventories.

Figure 8 shows the most popular scales in the considered papers. These scales are mainly used for evaluating the systems [e.g., Perceived Persuasiveness Scale (Drozd et al., 2012; Thomas et al., 2019), and susceptibility to persuasion (Modic et al., 2018)], or for assessing user types and identifying user groups [e.g., the Big Five Inventory (Goldberg, 1999), and the gamification User Type Hexad Scale (Tondello et al., 2016)]. Some of these scales are connected to the theories and models presented in Figure 8. For example, the Big Five Inventory is used to assess an individual's personality based on the Five Factor Model (McCrae and John, 1992). As Figure 8 shows, the Big Five Inventory (BFI) is the most popular scale. This is expected given that personality is the most popular aspect to identify individual differences (as described in section 4.3, Figure 6), and the BFI is the most common scale to evaluate the big five personalities. The “Other “ category include papers that are used by less than two papers, such as The Affective Usability Scale (AUS) (Orji et al., 2018a), Sport Orientation Questionnaire (O'Keefe, 2013), and Sports Motivation Scale (SMS-II) (Aldenaini et al., 2020).

Table 3 summarizes the personalization aspects along with their definitions and the most popular theories used under each aspect.

**Table 3:** Summary of personalization aspects and the most popular theories.

**Personalization aspect**	**Description**	**Models and theories**	**References**
Personality	*P*ersonality can be defined as “a set of characteristics possessed by a person that influence his or her cognitions, emotions, motivations, and behaviors in various situations” (O'Keefe, 2015)	• Five Factor Model (FFM) (McCrae and Costa, 1987) • Myers-Briggs Type Indicator assessment (MBTI) (McCrae and Costa, 1989) • Meta-judgmental measures of personality traits (Bassili, 1996)	Deci and Ryan, 2000; O'Keefe, 2013; Anagnostopoulou et al., 2019; Arden-Close et al., 2022; Bassili, 1996; Goldberg et al., 2006; Booth and Grant, 2009; Orji, 2016; Llagostera, 2012; Adaji, 2017; Feroz et al., 2021; Fogg, 2003; Cugelman, 2013; Barratt, 2017; Oyebode et al., 2021a; Goldberg, 1999; Oinas-Kukkonen and Harjumaa, 2009; Deterding, 2012; Alqahtani et al., 2022
Player type	This aspect is mostly used with persuasive games or gamified persuasive systems. Player types distinguish how different users interact with persuasive games or gamified persuasive elements. Many player type models have been developed	• BrainHex (Nacke et al., 2014) • Gamification User Type Hexad Framework (Tondello et al., 2016)	Forget et al., 2008; Martin and Kwaku, 2019; Alslaity and Tran, 2020b; Klock et al., 2020; Knowles et al., 2014; Foulonneau et al., 2016; Alslaity and Tran, 2020a
Motivational style	This aspect covers the types of motivations users have toward a particular behavior	• Sports Motivation Scale (SMS-II) (Pelletier et al., 2013) • Achievement Goal Questionnaire-Revised (AGQ-R) (Strunk, 2014) • Self-Determination Theory (SDT) (Deci and Ryan, 2000) • Motivational orientation (O'Keefe, 2013)	Alqahtani et al., 2022; Roosta and Taghiyareh, 2016; Sporrel et al., 2021; Spelt et al., 2022
Culture	This aspect considers users' models based on cultural differences	• Social Cognitive Theory (SCT) (Bandura, 2002) • Hofstede's cultural dimensions (Hofstede and Milosevic, 2018)	Jia et al., 2016; Orji et al., 2017a; Modic et al., 2018; Monteiro-Guerra et al., 2020; McCrae and Costa, 1987; Hofstede and Milosevic, 2018
Stages of change	This dimension considers users' difference based on their intention to change behavior	• Transtheoretical Model (TTM) (Heath, 2014)	Oyibo et al., 2019; Mulchandani et al., 2022; Oyebode and Orji, 2022
Cognitive ability	This aspect considers users' cognitive level as an important dimension for personalization	• Educational Testing Service (ETS) Kit of Referenced Test for Cognitive abilities (Schaie et al., 1991) • Intelligent Quotient (FSIQ scores) (Wiens et al., 1993) • Wechsler Abbreviated Scale Intelligence-II (Abdullahi et al., 2018, 2019b)

### 4.5. Technology platform

**Figure 9:**
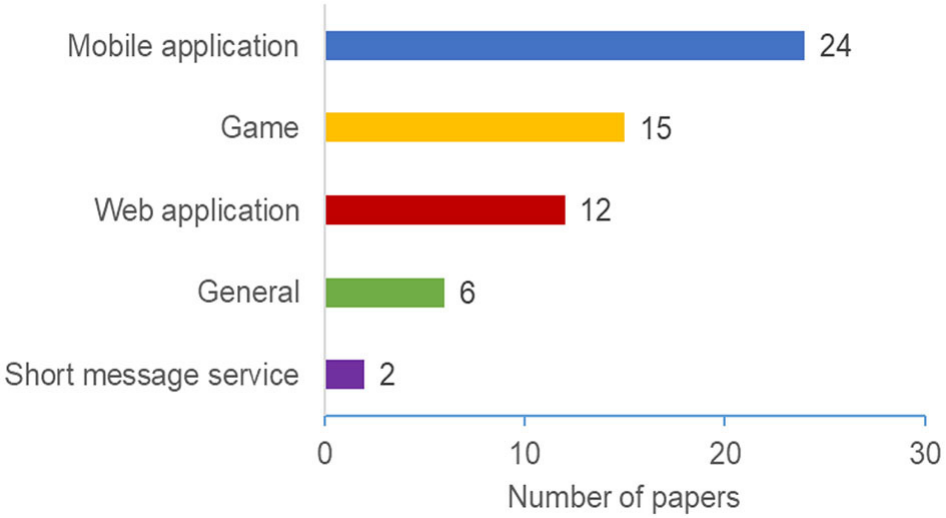
Technology platforms.

Persuasive interventions can be implemented in different forms using various technologies. Our review revealed four main types of technological platforms used for personalized persuasive interventions design, as depicted in Figure 9, and Table 4. Our study revealed that Mobile Apps are the most common technology used in the considered papers, followed by Games (15 papers), and Web Apps (12 papers). Some interventions are based on sending persuasive messages via Short Message Services (SMS) instead of developing a persuasive app. This type of personalized persuasive intervention is categorized under “Short Messages Service.” Only two papers used this type of technology. Finally, six papers discussed personalizing persuasive interventions without developing a system or a prototype. That is, they discussed and evaluated personalizing PT in general. For example, Abdullahi et al. (2019a) investigated how to tailor persuasive health interventions to individuals, by using survey methods to examine how user characteristics (e.g., different gender groups and age groups) are related to components of subjective wellbeing. We grouped these papers under the “General” category.

**Table 4:** Summary of papers by platform.

**Platform**	**References**
Mobile application	McCrae and Costa, 1987, 1989; Bandura, 2002; Fogg and Fogg, 2003; Midden et al., 2008; Booth and Grant, 2009; Fogg, 2009; Brynjarsdóttir et al., 2012; Deterding, 2012; Gardner et al., 2012; Matthews et al., 2016; Orji, 2016; Adaji, 2017; Orji et al., 2017b, 2019b; Altmeyer et al., 2018; Hofstede and Milosevic, 2018; Modic et al., 2018; Mora et al., 2019; Aldenaini et al., 2020; Alslaity and Tran, 2020a, 2021; Feroz et al., 2021; Mazeas et al., 2022; Oinas-Kukkonen et al., 2022
Game	Goldberg, 1999; O'Keefe, 2013; Heath, 2014; Busch et al., 2015; Anagnostopoulou et al., 2018; Orji and Moffatt, 2018; Orji et al., 2018b; Martin and Kwaku, 2019; Alslaity and Tran, 2020b; Klock et al., 2020; Oyebode et al., 2021b; Arden-Close et al., 2022; Ghorbani and Semiyari, 2022
Web application	Goldberg et al., 2006; Forget et al., 2008; Oinas-Kukkonen and Harjumaa, 2008; Gardner et al., 2012; Nacke et al., 2014; O'Keefe, 2015; Orji et al., 2017a; Abdullahi et al., 2018; Feroz et al., 2021; Alqahtani et al., 2022; Jankovič et al., 2022
General	Bassili, 1996; Jia et al., 2016; Jones and Simons, 2017; Abdullahi et al., 2019a; Monteiro-Guerra et al., 2020
Short messages	Fogg, 2002, 2003

### 4.6. Target outcome

**Figure 10:**
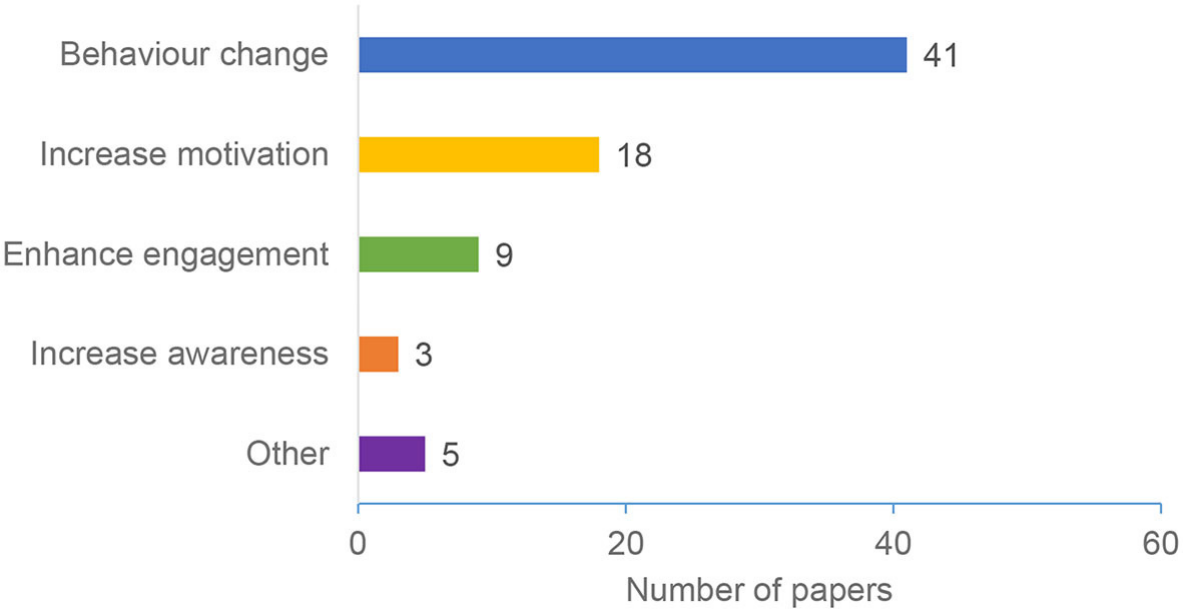
Target outcome.

Persuasive technologies are applied in various domains, and they have different goals or target outcomes. Target outcome is the intended goal of using the personalized persuasive intervention. Our study identified four main target outcomes: Behavior Change, Increase Motivation, Enhance Engagement, and Increase Awareness (Figure 10 and Table 5). Our results revealed that *Behavior Change* is the most popular target outcome, with 57% of the papers targeting this outcome. *Increase Motivation* comes next (25%), followed by *Enhance Engagement* (13%), and finally, *Increase Awareness* (4%). Some papers considered multiple goals (e.g., Increase Motivation and Enhance Engagement). As such, these papers were considered under both categories. Other target outcomes related to factors such as supporting calm breathing, increasing time spent using the app, and maintaining self-care. The “Other” target outcome category includes self-management (i.e., using the app to manage a disease like diabetes) (Gardner et al., 2012), supporting calm breathing (Oyebode et al., 2021a), changing attitude (Barratt, 2017), and increasing the learner's intended effort (Cugelman, 2013).

**Table 5:** Summary of target outcome references.

**Target outcome**	**References**
Behavior change	McCrae and Costa, 1987, 1989; Goldberg, 1999; Deci and Ryan, 2000; Bandura, 2002; Fogg, 2002, 2003, 2009; Goldberg et al., 2006; Oinas-Kukkonen and Harjumaa, 2009; Brynjarsdóttir et al., 2012; Deterding, 2012; Llagostera, 2012; O'Keefe, 2013; Knowles et al., 2014; Orji et al., 2014, 2019a,b; Foulonneau et al., 2016; Jia et al., 2016; Matthews et al., 2016; Adaji, 2017; Barratt, 2017; Abdullahi et al., 2018, 2019b; Altmeyer et al., 2018; Modic et al., 2018; Martin and Kwaku, 2019; Alslaity and Tran, 2020a, 2021; Monteiro-Guerra et al., 2020; Oyebode et al., 2021b; Alqahtani et al., 2022; Mazeas et al., 2022
Increase motivation	McCrae and Costa, 1987, 1989; Bassili, 1996; Forget et al., 2008; Midden et al., 2008; Oinas-Kukkonen and Harjumaa, 2008; Booth and Grant, 2009; Busch et al., 2015; Jones and Simons, 2017; Orji et al., 2017a, 2018b; Altmeyer et al., 2018; Orji and Moffatt, 2018; Abdullahi et al., 2019a; Jalowski et al., 2019; Martin and Kwaku, 2019; Aldenaini et al., 2020; Mulchandani et al., 2022
Enhance engagement	Midden et al., 2008; Oinas-Kukkonen and Harjumaa, 2008; Nacke et al., 2014; O'Keefe, 2015; Orji et al., 2017a,b; Anagnostopoulou et al., 2018; Alslaity and Tran, 2020b; Jankovič et al., 2022
Increase awareness	Heath, 2014; Hofstede and Milosevic, 2018; Ghorbani and Semiyari, 2022

### 4.7. Evaluation approach

This section discusses the evaluation approaches used in the considered papers. Particularly, this section discusses the following aspects: evaluation method, the target audience, study duration, number of participants, age distribution, and gender distribution.

#### 4.7.1. Evaluation method

**Figure 11:**
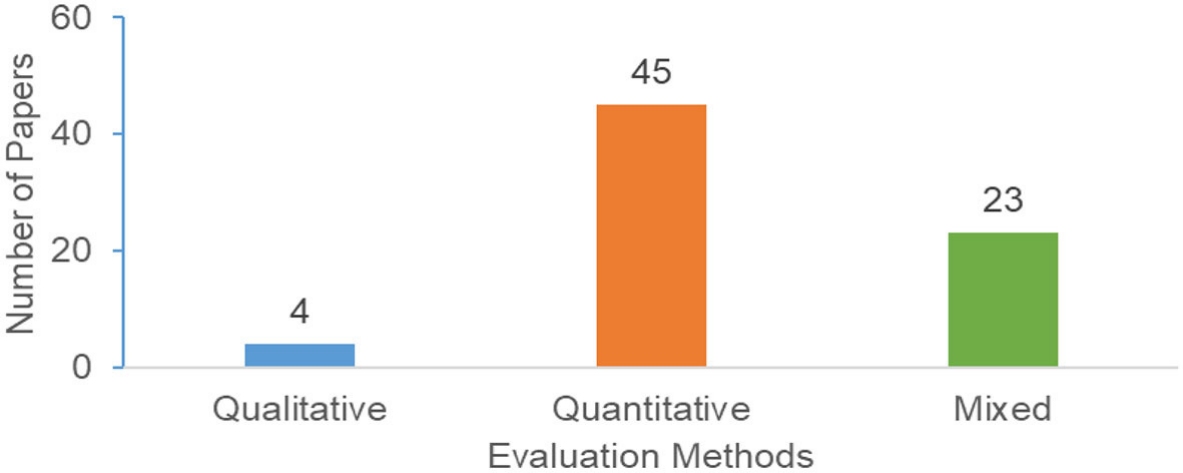
Evaluation method.

First, we explored the various kinds of evaluation methods used. From the 72 papers, three types of study methods were identified: quantitative, qualitative, and mixed. Quantitative methods employed include questionnaires (subjective ratings) and data logs (e.g., time spent and number of looks), whereas qualitative methods were think-aloud sessions and semi-structured interviews. Our results show that most of the studies used a quantitative evaluation method. As Figure 11 shows, 45 papers used a quantitative evaluation only, while 23 other studies used both quantitative and qualitative (a mixed-methods approach). That means 93% of the papers used the quantitative method. There were various ways in which a mixed methods approach occurred. For example, there were studies that began with a quantitative analysis of subjective responses gathered using closed-ended questions followed by an analysis of qualitative insights collected using open-ended questions based on written comments (Orji et al., 2018b; Oyebode et al., 2021b), while other studies report employing a qualitative approach (e.g., think-aloud session) followed by a quantitative evaluation (e.g., online questionnaire) (Senette et al., 2018). There were also some studies that conducted a quantitative study followed by semi-structured interviews with a subset of participants to gain additional insights (Mulchandani et al., 2022). On the other hand, qualitative methods are used in a total of 27 papers, out of which only four papers used qualitative methods only, while 24 papers used a mixed-methods approach.

#### 4.7.2. Target audience

**Figure 12:**
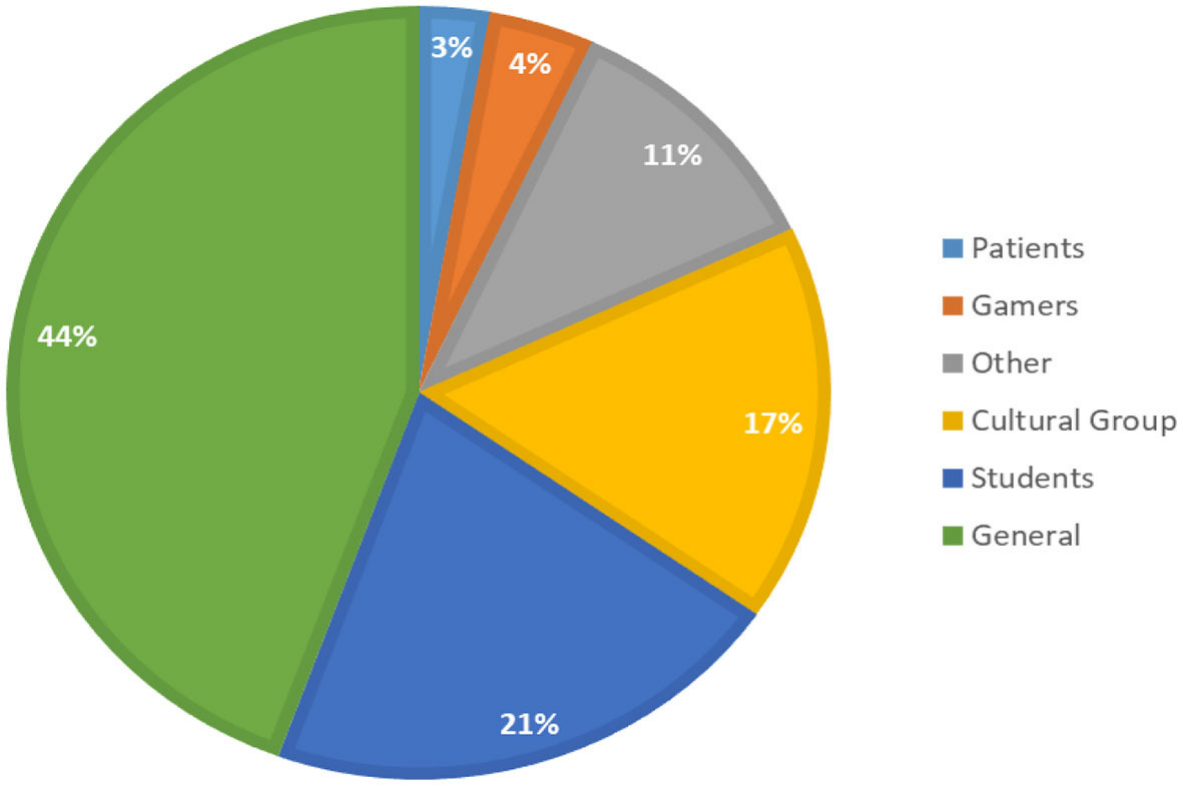
Distribution of papers by the audience.

Regarding the target audience, the reviewed papers consider various audience groups for evaluating their personalized persuasive intervention. Figure 12 summarizes the distribution of the most common audience. As the figure shows, 44% of the studies were conducted with the general audience (i.e., they do not target a particular group of audience, anyone can participate). The next most popular audience is students. This category includes various student categories, such as university, graduate, high school, etc. Some studies targeted a specific group from a specific geograpical location (e.g., Canada, Nigeria, Africa). We combined all these studies under one category called *Cultural Group*, with 17% of the papers. Some papers target gamers (4%), and patients (3%). These are the most popular audience groups. The remaining papers are grouped under a single category called “Others,” and it includes the least popular audience groups, such as computer programmers, drivers, healthy people, gym trainers, and gamification experts. “Others” is different than “General”; papers that are grouped under the “General” category are papers that do not have specific requirements for participants. On the other hand, “Other” are papers that target specific group of audience. These specific groups are not mentioned explicitly in the figure for clarity purposes.

#### 4.7.3. Study duration

**Figure 13:**
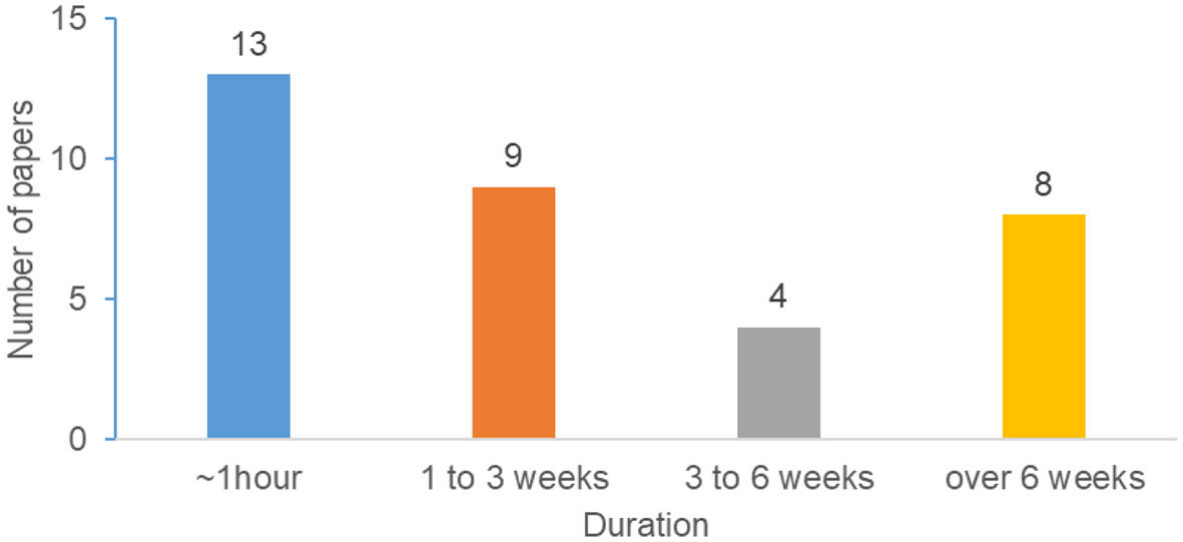
Distribution of papers based on study duration.

The reviewed papers conducted studies that lasted for various durations ranging from around an hour, to more than 6 weeks. We grouped the studies into four categories (about 1 h, 1–3 weeks, 3–6 weeks, and over 6 weeks). Out of 72 studies, only 34 papers mentioned the duration of the study. Figure 13 depicts the study duration results based on 34 papers. Many of the studies required <1 h, while only eight papers conducted studies for more than 6 weeks. A common limitation that is frequently reported in studies employing a short-term evaluation (e.g., 1-h laboratory sessions) is the need to conduct a longer-term study to evaluate the effectiveness of the persuasive intervention over a longer period.

For studies that evaluate the effectiveness of an intervention for over a week, results have been mixed.

## 5. Discussion

This section provides our insights and recommendations for designing personalized persuasive interventions based on the results obtained in our study. It also provides future research directions based on our observations through this review.

### 5.1. Insights

Following are the main insights and recommendations based on our results and the research questions:

*RQ1: What are the trends in the research in the domain of personalization and PT?*
  - Our results demonstrated an overall increase in the number of research in personalization persuasive and behavior change interventions (as shown in Figure 2). The majority of the papers were published in conferences and ACM was the most common publisher. The results also shows that education, General Health, and Physical Activity are the most common domains (Figure 5), Personality traits is the most considered personalization aspect (Figure 6), and the Five Factor Model is the mostly used model to capture personality types (Figure 7). Besides, there are higher number of mobile application interventions compared to other technologies, such as web applications and games (Figure 9), and the majority of the proposed interventions target at changing behavior toward a particular goal (Figure 10).
*RQ2: What are the personalization aspects used in persuasive interventions?*
*RQ3: How are individual differences captured in persuasive and behavior change interventions?*
  - Based on our analysis of individual differences, Section 4.3 showed that Personality trait is the most popular personalization aspect for understanding and identifying individual differences. Research has shown that personality traits represent a set of frequent interpersonal situations that construct patterns. These patterns are known as relatively consistent, and they do not change significantly during individuals' life (Sullivan, 2014). Thus, it is considered as one of the most efficient dimensions to model users and provide personalized interventions. Therefore, personality traits can be considered as a good option to build users' models and provide personalized persuasive interventions.
  - Individual differences can be captured using different ways relying mainly on theories and scales. The results presented in Section 4.4 identified various models and theories for individual differences. We noticed that the same personalization aspect can be captured using different scales. For example, the most common aspect, personality traits, can be captured using the Big Five Inventory (BFI) (Tupes and Christal, 1992), and the International Personality Item Pool (Goldberg et al., 2006). Although these scales have been adopted in the PT domain, there is no clear guidelines on which scale to use under which conditions. *Therefore, selecting the most appropriate scale and theory for a particular intervention is not a straightforward task, and designers should take this into consideration*.
*RQ4: What are the most common theories and practices that have been used for developing and evaluating personalized PT?*
  - Our results shows that researchers rely on several theories to capture the differences between users. The majority of these theories are adopted from the social sciences. The results (Figure 7) show that the most commonly adopted theories are Big five Personalities, the BrainHex Gamer Type, the Transtheoritical Model of Change, the Social Cognitive Theory, the Self-Determination theory, Fogg's Behavioral Models, the Health Belief Model, and the ARCS Model of Motivation.
  - Regarding evaluating personalized persuasive technologies, our results show that qualitative methods are used in a total of 27 papers, out of which only four papers used the qualitative method alone, while 24 papers used a mixed-methods approach. This indicates that most of the studies in this area use quantitative methods. This can be due to the simplicity of conducting quantitative studies compared to qualitative studies, as qualitative research (e.g., interviews) can be rather time-consuming.
*RQ5: What are the most common challenges and Limitations facing the domain of personalized and adaptive systems?*
  - Our results show discrepancies in reporting various aspects of the conducted studies. For example, out of 72 studies, 38 papers did not mention the duration of the study. Also, several studies did not mention the age or gender details. For example, some studies only mentioned the average age of the participants.
  - Another important point related to the studies and evaluation is that many papers reported results based on a short-term study, <3 weeks (as mentioned in Section 4.7.3). This issue limited the generalizability of the results.
  - Also, we noticed that most of the studies use either a prototype or an app developed for evaluation purposes only. That is, there is a lack of studies that evaluate the personalization approaches in-the-wild.
  - Related to the previous point, we also found that most of the papers consider fixed personalization rather than automatically adaptive personalization.
*RQ6: What are the most common goals of personalized persuasive interventions?*
  - Our study results (Section 4.6) revealed personalized PTs target various goals. The four major categories of these goals are Behavior Change, Increase Motivation, Enhance Engagement, and Increase Awareness, and behavior change is identified as the most common goal. Most of the considered papers target one goal, such as O'Keefe (2015), Adaji (2017), and Orji and Moffatt (2018). However, some papers target several goals, such as increasing motivation and enhancing engagement (Oinas-Kukkonen and Harjumaa, 2008). Our study also identified other goals which are less common, including managing a disease (e.g., diabetes) (Gardner et al., 2012), supporting calm breathing (Oyebode et al., 2021a), changing attitude (Barratt, 2017), and increasing the learner's intended effort (Cugelman, 2013).

The next section answers the last research question (*RQ7: What are the limitations and future research directions in the domain of personalized persuasive interventions?)*.

### 5.2. Recommendations

This section discusses our recommendations, which are based on the study results and can be summarized by the following points:

- The popularity of the theories and scales (Figure 7) does not mean they are the dominant theories for all personalized persuasive interventions; The selection of these theories depends on different factors, including the domain area, intervention requirements, target users, and more. For example, if we aim to design a personalized persuasive health system that provides interventions based on user's' awareness of a health issue, then the Health Belief model would be a suitable theory to capture users' differences (Alslaity and Tran, 2021). Besides, users' differences can be captured based on multiple factors and theories. Intuitively, using more theories and collecting more information about users would enhance personalization and provide a more accurate personalization mechanism. However, collecting more information complicates the personalization process, requires more effort, and may cause users' resistance due to time, effort, and privacy concerns. Therefore, *selecting the personalization aspect and corresponding theories is an essential task that needs to be considered carefully when designing a persuasive intervention*.
- In general, it is much easier to recruit participants for quantitative studies. Nonetheless, research has shown that it is beneficial to use qualitative data to support quantitative results (Guetterman et al., 2015). Qualitative results provide more fine-grained thoughts and opinions that can be a good compliment to further support or elaborate on the quantitative results (Sandelowski, 2000). Therefore, *our suggestion is to employ mixed methods to get a more insightful and fine-grained evaluation of personalized PT*.
- Not presenting sufficient details about studies has a negative impact on the replicability and repeatability of studies, which, in turn, degrades the progress in the research and development of personalized persuasive interventions. Thus, *we suggest that when reporting empirical research, include the duration of the intervention, as well as age and gender information so that other researchers can build on existing work*.
- Persuasive technologies are used widely for behavior change (as shown in Section 4.6). It is known that behavior change and habit formation require a long-time process [e.g., in the health domain, research suggests that it takes at least 10 weeks (2–3 months) for habit formation (Gardner et al., 2012)] that cannot be assessed in a very short term. Likewise, Anagnostopoulou et al. (2018) also identified a lack of large-scale and longitudinal evaluations in the field. Accordingly, *we recommend conducting longitudinal studies to evaluate the long-term impact of the interventions*.
- *We urge the use of in-the-wild and long-time studies* as they would help establish the long-term influence of PT in promoting desirable changes. It would also help to continue following the users as when the system detects that an intervention is ineffective or plateaus, the system can offer motivation that is personalized to help the user continue working on changing or improving their behaviors.
- Oinas-Kukkonen et al. (2022) argue that because a user's behavior can change over time, the grounds for personalization may change without the system detecting the change, which results in an “outdated” view of the user. The more personalized the intervention, the more users relate to it and find it useful. This, in turn, will increase the likelihood of adoption of the intervention. However, recent research shows that real-time physical activity coaching systems employ rather simple forms of personalization, such as Feedback, Goals Setting and User Targeting, while very few systems include adaptation, context awareness, or self-learning techniques (Monteiro-Guerra et al., 2020). Thus, we suggest that *it is important to design interventions that continuously monitor the user so that the system is kept up-to-date about the user's habits and motivation*. This way, the system can adapt to the user and increase the likelihood that the user will be successful at achieving the target behavior. Based on previous studies showing that personalized recommendation using a combination of gamification and continuous player modeling would increase users engagement and motivation (Zhao et al., 2020), we also *recommend integrating a combination of personalization techniques into the design of PT* to increase its effectiveness for motivating behavior change.

### 5.3. Future direction

Many questions remain unanswered in the field on how we can further explore and better personalize PT to motivate behavior change. Below are some potential research questions provoked by the recommendations above as researchers continue to investigate different strategies, theories, individual differences, personalization techniques, and methods to motivate behavior change. We offer the following three general areas for further investigation: (1) improve the experience of educational games and general health solutions, (2) compare the effectiveness of various models, and (3) the pervasiveness of mobile applications and solutions.

Firstly, we observed that one of the most popular domains studied is the domain of education and general health. This suggests that these domains are capturing much research attention, and it is perhaps traditionally these are domains that examine human motivation—motivate learning and motivate the adoption of a healthier lifestyle. In particular, edutainment in the classroom uses games and videos (which are forms of technology) to motivate learning, and thus, it is expected that it will continue to be a popular topic of study as teachers are constantly finding new ways to motivate student learning. Below are some potential research questions:

- What gamification elements can be combined to increase the effectiveness of personalization?
- What design improvements can be made to adjust the personalization of the student's learning to increase the effectiveness of the intervention?
- Can gamification elements be adjusted or removed as the behavior gradually forms into a habit?

Secondly, we observed that although there were some studies that utilized multiple models/theories, many studies only employed a single behavioral model. Combining and comparing the effectiveness of related theories/models (e.g., player type models such as the BrainHex and Hexad) can give insight into refining the models and gain a more complete understanding of how to tailor the persuasive system to user's preferences. It is expected that this will also be on the research agenda for many researchers as novel systems (e.g., games and mHealth apps) are being designed and tested to personalize the user experience and motivate behavior change. Below are some potential research questions:

- What models/theories can be combined to provide a better picture of how to tailor the design of persuasive/gamified systems?
- What other models/theories can be used to inform the design and enhance the adoption of personalized persuasive systems?
- How can theories/models be improved to better understand what motivates behavior change and increase the effectiveness of personalizing persuasive systems?
- Are some models/theories more useful for understanding personalization, user motivations, and behaviors compared to others?

Thirdly, one more area we identified that is likely to continue growing and will be heavily researched is mobile solutions due to their ubiquitous nature. Many conference and journal venues that were part of this review consisted of a mobile component (e.g., novel applications for mobile and ubiquitous gaming, entertainment, networking, and advertising, as well as social implications of mobile and ubiquitous multimedia systems) as a core topic of interest. Thus, it is likely to be a topic of interest for many as researchers continue to investigate how to design and personalize persuasive systems that can fit into a mobile context. Below are some potential research questions:

- What other application domains can take advantage of mobile technologies and make persuasive systems more effective?
- Can mobile technologies offer a more integrated experience by collecting user data such as habits and preferences and generate more personalized recommendations?

Also, there is a need for domain-specific studies that focusses on the most suitable settings for each domain, considering the relationship between application domain and other factors, such as target outcome, personalization aspect, theories and models, technology and platforms, etc., such studies serve as references for developing personalized persuasive interventions that are adapted to different domains.

## 6. Conclusion

In closing, research on personalizing persuasive technology is growing. This is to address the limitations of the “one-size-fits-all” approach. In addition to personalization, many researchers are investigating the effectiveness of combining related concepts such as gamification and player/user modeling, along with various forms of technologies ranging from mobile to AR/VR.

In this review, our aim was to examine the existing literature concerning personalization and individual differences in persuasive and behavior change intentions. Thus, we conducted a systematic review of literature that has been published within the past 10 years. Based on our results, we found that persuasive technologies are used widely for behavior change, and personality is the most popular characteristic that has been researched and applied. We also found that there is a lack of longitudinal studies as most papers reported results based on a short-term evaluation (<3 weeks) and that most studies employed a quantitative approach.

As persuasive techniques and strategies continue to be applied to developing interventions that motivate behavior change, there will be a variety of ways to personalize the system design. This creates many opportunities for technology to make itself into people's everyday life and influence their choices and behaviors. Thus, it is important to understand how to better design these systems, making them effective, motivational, and personal to increase the likelihood of adoption.

## Author contributions

AA: conceived and designed the research, analyzed the data, prepared figures and/or tables, authored or reviewed drafts of the paper, and approved the final draft. GC: analyzed the data, prepared figures and/or tables, reviewed drafts of the paper, and approved the final draft. RO: authored or reviewed drafts of the paper and approved the final draft. All authors contributed to the article and approved the submitted version.

## Appendix I

**Table A1:** Search query.

**Resource**	**Query**
***Scopus***	**(KEY (*adapt** OR *personali** OR *individual*) AND KEY (*persuas** OR *motivat** OR *convinc** OR *influenc**) AND KEY (*intervent** OR *app** OR *solut** OR *system* OR *technology*)) AND (LIMIT-TO (PUBYEAR, *2022*) OR LIMIT-TO (PUBYEAR, *2021*) OR LIMIT-TO (PUBYEAR, *2020*) OR LIMIT-TO (PUBYEAR, *2019*) OR LIMIT-TO (PUBYEAR, *2018*) OR LIMIT-TO (PUBYEAR, *2017*) OR LIMIT-TO (PUBYEAR, *2016*) OR LIMIT-TO (PUBYEAR, *2015*) OR LIMIT-TO (PUBYEAR, *2014*) OR LIMIT-TO (PUBYEAR, *2013*) OR LIMIT-TO (PUBYEAR, *2012*))**
*Pubmed*	((“adapt*”[Title] OR “Personali*”[Title] OR “customiz*”[Title] OR “Individual”[Title]) AND (“Persuas*”[Title] OR “Behav*”[Title] OR “motiv*”[Title] OR “convinc”[Title] OR “influenc*”[Title]) AND (“Intervention”[Title] OR “App”[Title] OR “solution” [Title] OR “system”[Title]) •(((((adapt*[Title]) OR (“Personali*”[Title]) OR (“customiz*”[Title]) OR (“Individual”[Title])) AND (“Persua*”[Title] OR “motiv*”[Title] OR “convinc*”[Title] OR “influenc*”[Title])) AND (“Intervent*” OR “App*” OR “solution” OR “system” OR “Technology”[Title])
*ACM*	“query”: { Abstract:(adapt* OR personaliz* OR customiz* OR individual*) AND Abstract:(persua* OR motiv* OR convinc* OR influenc*) AND Abstract:(intervent* OR app* OR solut* OR system*) } “filter”: { Publication Date: (01/01/2012 TO 10/31/2022)
*IEEE*	(“Index Terms”:“adapt*” OR “Index Terms”:“Personali*” OR “Index Terms”:“customiz*” OR “Index Terms”:“Individual”) AND (“Index Terms”:“Persua*” OR “Index Terms”:“motiv*” OR “Index Terms”:“convinc*” OR “Index Terms”:“influenc*”) AND (“Index Terms”:“Intervent*” OR “Index Terms”:“App*” OR “Index Terms”:“solution” OR “Index Terms”:“system” Or “Technology”)
